# Relationships between barley consumption and gut microbiome characteristics in a healthy Japanese population: a cross-sectional study

**DOI:** 10.1186/s40795-022-00500-3

**Published:** 2022-03-14

**Authors:** Tsubasa Matsuoka, Koji Hosomi, Jonguk Park, Yuka Goto, Mao Nishimura, Satoko Maruyama, Haruka Murakami, Kana Konishi, Motohiko Miyachi, Hitoshi Kawashima, Kenji Mizuguchi, Toshiki Kobayashi, Hiroshi Yokomichi, Jun Kunisawa, Zentaro Yamagata

**Affiliations:** 1 Research and Development Department, Hakubaku Co., Ltd., 4629, Nishihanawa, Chuo, Yamanashi, 409-3843 Japan; 2 Department of Health Sciences, School of Medicine, University of Yamanashi, 1110, Shimokato, Chuo, Yamanashi, 409-3898 Japan; 3 Laboratory of Vaccine Materials, Center for Vaccine and Adjuvant Research and Laboratory of Gut Environmental System, National Institutes of Biomedical Innovation, Health and Nutrition, 7-6-8, Saito-Asagi, Ibaraki, Osaka 567-0085 Japan; 4 Artificial Intelligence Center for Health and Biomedical Research, National Institutes of Biomedical Innovation, Health and Nutrition, 7-6-8, Saito-Asagi, Ibaraki, Osaka 567-0085 Japan; 5 Department of Physical Activity Research, National Institutes of Biomedical Innovation, Health and Nutrition, 1-23-1, Toyama, Shinjuku-ku, Tokyo 162-8636 Japan; 6 Laboratory of Computational Biology, Institute for Protein Research, Osaka University, 3-2, Yamadaoka, Suita, Osaka 565-0871 Japan; 7 Department of Microbiology and Immunology, Kobe University Graduate School of Medicine, 7-5-1, Kusunoki-cho, Chuo-ku, Kobe, Hyogo 650-0017 Japan; 8 Graduate Schools of Medicine, Graduate Schools of Pharmaceutical Sciences, Graduate Schools of Science, and Graduate Schools of Dentistry, Osaka University, 1-1, Yamadaoka, Suita, Osaka 565-0871 Japan; 9 Department of Microbiology and Immunology and International Research and Development Center for Mucosal Vaccines, The University of Tokyo, 4-6-1, Shirokanedai, Minato-ku, Tokyo 108-8639 Japan; 10 Research Organization for Nano and Life Innovation, Waseda University, 513, Waseda-tsurumaki-cho, Shinjuku-ku, Tokyo 162-0041 Japan

**Keywords:** Barley, Beta-glucan, Microbiome, *Bifidobacterium*, *Butyricicoccus*

## Abstract

**Background:**

Barley contains abundant soluble beta-glucan fibers, which have established health benefits. In addition, the health benefits conferred by the gut bacteria have attracted considerable interest. However, few studies have focused on the barley consumption and gut bacteria of the Japanese population. In this study, we aimed to identify the relationship between the barley consumption and gut bacteria composition of the Japanese population.

**Methods:**

In total, 236 participants were recruited in Japan, and 94 participants with no complications of diabetes, hypertension, or dyslipidemia were selected for the study. We analyzed fecal samples from the participants, their medical check-up results, and responses to questionnaires about dietary habits. The participants were grouped according to their median barley consumption. Then, we assessed the relative abundance of 50 genera. Characteristic bacteria were evaluated for their relationship with barley consumption by multiple regression analysis, adjusted for disease and dietary habits, in all participants. We also analyzed the networks and clustering of the 20 selected genera.

**Results:**

According to the comparison between barley groups, *Bifidobacterium*, *Butyricicoccus*, *Collinsella*, *Ruminococcus* 2, and *Dialister* were characteristic candidate bacterias of the group that consumed large amounts of barley (*P* < 0.05). The relationship between barley consumption and *Bifidobacterium* remained after adjusting for disease and dietary habits, and that of *Butyricicoccus* remained after adjusting for disease. Furthermore, network and cluster analyses revealed that barley consumption was directly correlated with *Bifidobacterium* and *Butyricicoccus*.

**Conclusions:**

Barley consumption generates changes in the intestinal bacteria of the Japanese population. We found that *Bifidobacterium* and *Butyricicoccus* abundance was positively associated with barley consumption.

**Supplementary Information:**

The online version contains supplementary material available at 10.1186/s40795-022-00500-3.

## Background

The gut microbiome is important for health, and thus, several investigations have focused on the human gut microbiome. It has been reported that diet can alter the gut microbiome [1]. Microbiota-accessible carbohydrates, such as dietary fiber, are resistant to digestion in the small intestine and enter the large intestine undigested; therefore, they are likely to improve host metabolism [2].

Barley is an important cereal that contains the soluble fiber beta-glucan [3]. Barley improves metabolic dysfunction, increases the diversity of the gut bacteria, and increases bacteria such as *Blautia* [4]. Additionally, barley lowers postprandial blood glucose in healthy [3] and diabetic patients [5] and lowers cholesterol concentrations in Japanese people with mild metabolic syndrome after 12 weeks of consumption [6]. Therefore, barley consumption has many potential benefits to global health.

Barley consumption affects the gut bacteria and host health [4, 7, 8]. For example, a crossover study in Sweden found that barley intake increases blood concentrations of butyric acid (produced by intestinal bacteria) and decreases postprandial hyperglycemia [7]. Research in the USA found that barley intake increases the gut bacterial diversity and *Blautia* abundance and improves host cholesterol levels [4]. Another Swedish study found that barley intake promotes a high ratio of *Prevotella*/*Bacteroide*s and improves host blood glucose metabolism [8]. These results suggest that barley might beneficially modulate the composition of the gut bacteria and improve host metabolic health.

Indeed, the effects of barley on the gut bacteria have been established in several countries [4, 7, 8]. Furthermore, as the gut bacteria is affected by dietary habits and genetic factors, it is important to evaluate its effectiveness in different populations [9]. The Japanese gut bacteria is characterized by abundant *Blautia* and *Bifidobacterium*, which utilize the dietary fiber from seaweed, a common item in the Japanese diet [1]. Barley is the fourth most widely produced grain in the world, but its use as a food is limited as most of it is used for livestock feed and brewing materials, such as for beer. Japanese and Korean people, as well as some ethnic groups in Central Asia, have a long tradition of eating barley. However, there have only been a few reports on barley and intestinal bacteria in the Japanese population. Most studies on barley and the microbiome have regarded exhaled hydrogen concentrations as an alternative measurement of intestinal bacteria [7, 10, 11]; however, few studies have analyzed the relationship between the gut bacteria and barley consumption. Therefore, we aimed to define the relationship between barley and gut microbiome in healthy Japanese adults using next-generation sequencing.

## Methods

### Study design

The study was approved by the Yamanashi University Ethics Committee (approval No. 1824), the National Institutes of Biomedical Innovation, Health, and Nutrition Ethics Committee (approval No. 169–04), and the Chiyoda Paramedical Care Clinic Ethics Committee (approval No. 15000088). This study was conducted in accordance with the Declaration of Helsinki (2013) and was based on a registered study (UMIN000033479). Cross-sectional data were evaluated from the first year of the study to obtain an exploratory overview of the gut microbiome of a population that consumes barley. Sampling was conducted from August 2018 to March 2019.

We enrolled 272 participants, which were employees of the barley processing company Hakubaku Co., Ltd. Our target sample included at least 100 participants. We excluded those with disorders (Risk 2) and pre-disorders (Risk 1) of diabetes, hypertension, and dyslipidemia from the main analysis. Although gastrointestinal disorders were not included in the exclusion criteria, none of the participants had a history of ulcerative colitis or Crohn’s disease. There were also four participants with a history of irritable bowel syndrome, but none were undergoing treatment. Details of the exclusion criteria for disorders are shown in Table S1 (see Additional file 1). We classified the participants into two groups based on their median barley consumption rate (high, 3.5–28 and low, 0–3.5 g/1000 kcal).

### Measurements

The primary outcome was the association between barley consumption and the alpha-diversity of the microbiome, and the secondary outcome was the abundance of the 50 dominant genera sorted by mean relative abundance. We collected a copy of the participants’ medical check-up results. The participants’ medical check-ups were conducted at different hospitals, and measurements of body measurements, blood pressure, and biochemical markers were obtained. Blood pressure was measured at rest in a sitting position. Blood samples were taken after fasting for more than 7 h and measured with a calibrated measuring device. Body mass index (BMI) was calculated by dividing the weight by the square of the height. Dietary habits other than barley consumption were assessed using a brief self-administered diet history questionnaire (BDHQ; Gender Medical Research, Inc., Tokyo, Japan). Barley consumption (g/1000 kcal) was calculated using a questionnaire and the daily energy value from the BDHQ. Rice bowl size (200, 160, 140, and 100 g), proportion of barley mixed with white rice (0, 5, 10, 15, 30, and 50%), barley-mixed rice consumed per month (0, 0.5, 1, 4, 8, and 16 days/month), and barley consumption rate (g/d) were determined. Medical history, including medication (especially during the month of sampling), and consumption of fermented foods and supplements were determined using questionnaires.

### DNA extraction and 16S rRNA gene amplicon sequencing

Fecal samples were collected at home with guanidine thiocyanate (GuSCN) solution, and DNA was extracted and stored at 10–30 °C for up to 30 d [12]. Briefly, 0.2 mL of fecal samples, 0.3 mL of No. 10 lysis buffer (Kurabo Industries Ltd., Osaka, Japan), and 0.5 g of 0.1 mm glass beads (WakenBtech Co., Ltd., Tokyo, Japan) were homogenized using a PS1000 Cell Destroyer (Bio Medical Science, Tokyo, Japan) at 4260 rpm for 50 s at 25 °C. The homogenate was centrifuged at 13000×*g* for 5 min at 25 °C, and the DNA was extracted from the supernatant using a Gene Prep Star PI-80X automated DNA isolation system (Kurabo Industries Ltd). DNA concentration was determined with the ND-1000 NanoDrop Spectrophotometer (Thermo Fisher Scientific Inc., Waltham, MA, USA). The samples were stored at − 30 °C. The 16S rRNA gene was amplified from fecal DNA and sequenced [12]. The V3–V4 region of the 16S rRNA gene was amplified using the following primers (5′→3′): TCGTCGGCAGCGTCAGATGTGTATAAGCGACAGCCTACGGGNGGCWGCAG and GTCTCGTGGGCTCGGAGATGTGTATAAGAGACAGGACTACHVGGGTATCTAATCC. The DNA library for Illumina MiSeq was prepared using Nextera XT Index Kit v2 Set A (Illumina Inc., San Diego, CA, USA), and its concentration was determined with the QuantiFluor dsDNA System (Promega Corp., Madison, WI, USA). The 16S rRNA gene was sequenced using Illumina MiSeq (Illumina) as described by the manufacturer.

### Bioinformatics analysis

The sequence reads from Illumina MiSeq were analyzed using the Quantitative Insights Into Microbial Ecology (QIIME) software package (version 1.9.1) [13]. We used QIIME Analysis Automating Script (Auto-q) [14] to proceed from trimming paired-end reads to operational taxonomic unit (OTU) selection. We used open-reference OTUs picked with the UCLUST software against the SILVA (version 128) reference sequence to select OTUs based on sequence similarity (> 97%). The taxonomy (phylum, class, order, family, and genus) and relative abundance were calculated using the SILVA database (version 128) [13, 14]. The intestinal bacteria was compared based on 10,000 randomly selected reads per sample.

### Statistical analyses

#### Calculation of alpha-diversity

Data were exported as BIOM files and imported into R (version 3.6.0). Diversity was analyzed using the phyloseq R-package. Alpha-diversity indices of observed OTUs, Chao1, Shannon, and Simpson indices were calculated using the estimate_richness function.

#### Comparison of barley groups

To compare the results of the medical check-ups and dietary habits between the high and low barley groups, we used Student’s *t*-test. The alpha-diversity and relative abundance of each genus were analyzed using Mann-Whitney *U*-test. *P* values were adjusted using false discovery rate (FDR) methods. To confirm the reliability of the analyses, we explored the relationship between barley consumption groups (0 = low and 1 = high) and each bacteria using multiple regression analyses with all participants. We expressly set the amounts of *Bifidobacterium*, *Butyricicoccus*, *Collinsella*, *Ruminococcus* 2, and *Dialister* as outcomes. We adjusted the model for age, sex, risk of diabetes, dyslipidemia, and hypertension for model 1. In addition to model 1, we adjusted the model for consumption rate (g/1000 kcal) of cereals, sugar and sweetener, legumes, and beverages for model 2 and for cereals, sugar and sweetener, legumes, beverages, green vegetables, other vegetables, fish, and confectionery for model 3. We used the vif function of the car R-package to evaluate variance inflation factors (VIFs). All VIFs were < 5 and considered acceptable for these analyses [15].

#### Principal coordinate analysis of gut bacteria

We classified the participants into enterotypes A, B, and C using the pam function of the cluster R-package. We then summarized the composition of the intestinal bacteria by principal coordinate analysis (PCoA) using the vegdist function of the vegan R-package and the quasieuclid and dudi.pco functions of the ade4 R-package. Data were calculated using the Bray–Curtis method. Subsequently, the environmental factor arrows were fit to the PCoA figure using the envfit function of the vegan R-package. Significant genera were assessed using permutations of environmental variables.

#### Network analysis of significant bacteria and barley

To visualize the associations between barley and 20 genera selected based on a *P* < 0.1, we implemented a network analysis. The network is shown with lines of correlation with |*r*| > 0.15 (Kendall rank correlation tests). Different colors on the plots indicate different community groups. We fit the correlation data frame to the cc.df function of the igraph R-package using the reshape2 R-package and calculated bacteria community groups using the leading.eigenvector.community function. All analyses were carried out in R (version 3.6.0), and tests were two-sided; *P* < 0.05 was considered significant. All graphs except for those from the network analysis were created with the ggplot2 R-package [16].

## Results

### Participants

**Fig. 1:**
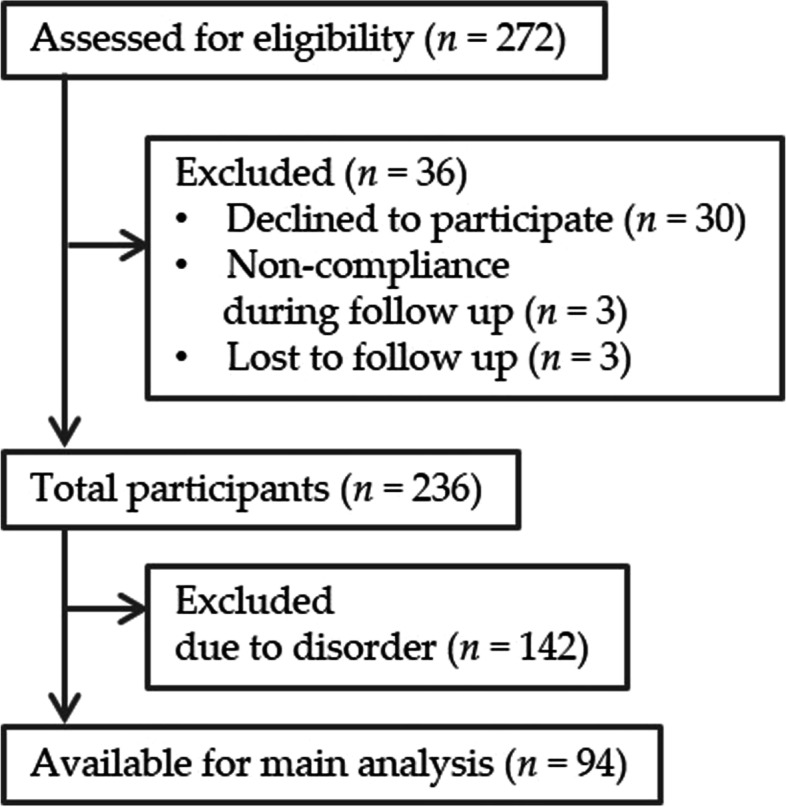
Flow chart of participants. Disorder means risk of diabetes, hypertension, and dyslipidemia

We obtained informed consent from 272 individuals, of which 30 participants resigned and six were excluded because of non-compliance and data loss. Ninety-four participants had no disorders and were included in the main analyses, and 236 were included in the multiple regression analyses (Fig. 1). Additionally, of 236 participants, four, six, and 10 participants consumed antibiotic drugs, laxative drugs, and drugs or supplements for controlling intestinal function (prebiotics and probiotics), respectively, 1 month prior to fecal sampling. We included these participants in the analyses because of the small number of people and the lack of regular intake.

The characteristics of the barley groups did not significantly differ (Table 1). However, fasting glucose concentrations tended to be higher in the high barley group (*P* = 0.054). Disorder risk did not significantly differ between the groups (Table S1, see Additional file 1). The mean age of the males was higher than females (males, 43 ± 12 [mean ± SD] years; females, 37 ± 11 years; *P* < 0.001), and therefore, a male had a higher risk of disease and a higher percentage was excluded. Table 2 and Table S2 show the differences in dietary habits between the barley groups. The barley consumption was 1.5 ± 1.2 g/1000 kcal and 7.7 ± 4.8 g/1000 kcal in the low and high barley groups, respectively. The consumption rates of dietary fiber (*P* < 0.001), cereals (*P* = 0.048), sugar and sweetener (*P* = 0.03), and beverages (*P* = 0.047) were significantly different between the barley groups, and those of legumes (*P* = 0.056) tended to be higher in the high barley group. Therefore, we selected these four dietary categories for adjustment in the multiple regression analyses (model 2). Additionally, green leafy vegetables, carrot and pumpkin in the green vegetable category, boiled fish in the fish category, and Japanese confectionery and ice cream in the confectionery category had a significant or slight difference between the barley groups. Moreover, seaweed and mushroom, which are known to contain soluble fiber, were in the “other vegetable” category. Therefore, we added these four variables to model 2 and conducted multiple regression analyses (model 3).

**Table 1:** Characteristics of the participants in the barley groups

	Total (*n* = 94)	Low barley (*n* = 47)	High barley (*n* = 47)	
Variable	0–28 g/1000 kcal^ a^	0–3.5 g/1000 kcal^ a^	3.5–28 g/1000 kcal^ a^	*P* ^b^
Male, *n* (%)	54 (57%)	32 (68%)	22 (47%)	
Age (years)	36 ± 10	36 ± 10	36 ± 11	0.82
Body mass index (kg/m^2^)	21.5 ± 3.4	21.3 ± 4.1	21.6 ± 2.5	0.67
Systolic pressure (mmHg)	111 ± 11	110 ± 11	112 ± 11	0.25
Diastolic pressure (mmHg)	68 ± 9	67 ± 9	69 ± 8	0.39
Fasting glucose (mg/dL)	86 ± 7	84 ± 7	87 ± 7	0.054
HbA1c (%)	5.3 ± 0.2	5.3 ± 0.2	5.3 ± 0.2	0.26
TG (mg/dL)	69 ± 28	67 ± 30	71 ± 26	0.42
HDL-cholesterol (mg/dL)	65 ± 13	66 ± 13	63 ± 12	0.26
LDL-cholesterol (mg/dL)	95 ± 15	93 ± 14	97 ± 16	0.15

Data are shown as means ± SD or *n* (%)

^a ^Range of barley consumption

^b ^Student’s *t*-test

**Table 2:** Comparison of diet between the barley groups

	Low barley (*n* = 47)	High barley (*n* = 47)	
Variable	0–3.5 g/1000 kcal ^a^	3.5–28 g/1000 kcal ^a^	*P* ^b^
*Daily Nutrition*			
Energy (kcal)	1693 ± 456	1765 ± 562	0.50
Protein (g/1000 kcal)	35 ± 6	34 ± 6	0.58
Fat (g/1000 kcal)	30 ± 6	30 ± 6	0.85
Carbohydrate (g/1000 kcal)	130 ± 20	129 ± 18	0.75
Dietary fiber (g/1000 kcal)	4.9 ± 1.3	6.1 ± 1.7	< 0.001
Na (mg/1000 kcal)	3608 ± 948	3871 ± 1094	0.22
*Dietary habits (g/1000 kcal)*			
Barley	1.5 ± 1.2	7.7 ± 4.8	< 0.001
Cereal	213 ± 55	236 ± 58	0.048
Legume	20 ± 15	27 ± 20	0.06
Green vegetable	37 ± 23	44 ± 32	0.2537
Other vegetable	62 ± 29	72 ± 45	0.2001
Fruit	36 ± 32	35 ± 30	0.87
Fish	32 ± 15	31 ± 16	0.74
Sugar and sweetener	3.9 ± 3.7	2.4 ± 2.4	0.03
Confectionery	26 ± 20	25 ± 17	0.81
Beverage	439 ± 271	335 ± 226	0.047

Data are shown as means ± SD

^a ^Range of barley consumption

^b^ Student’s *t*-test

### Enterotypes

**Fig. 2:**
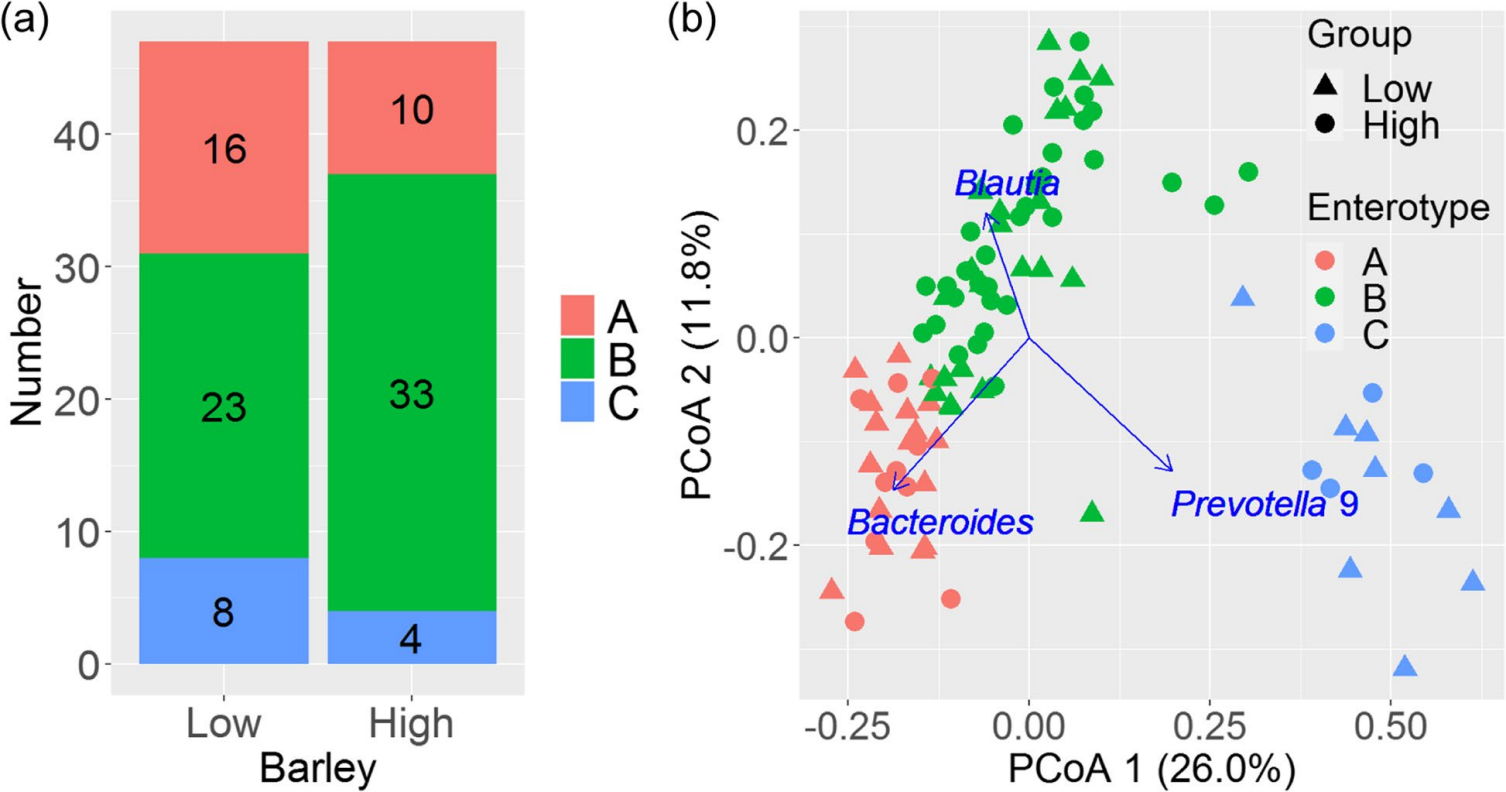
Microbiome enterotypes in high and low barley groups (*n* = 94) aged 19–65 years in 2018. **a** Comparison of numbers of enterotypes A, B, or C in each group. **b** Plot of principal coordinate analysis (PCoA). Color indicates enterotype; symbols indicate high or low barley consumption. Arrows indicate the top three environmental factors

Figure 2a describes the number of participants with each enterotype. Enterotype B was dominant and enterotypes A and C were less abundant in each group. The numbers of each enterotype did not significantly differ between the groups (*P =* 0.20). Figure 2b shows an overview of the PCoA of the microbiome. The distribution of the plots was laid out in a “∧” shape and separated into three groups. Clusters A and B were distributed in the negative direction of PCoA1 and cluster C in the positive direction. Clusters A and B were divided by PCoA2, and cluster B was in an intermediate position between A and C. The driven genera in each enterotype were *Bacteroides* (A), *Blautia* (B), and *Prevotella* 9 (C) (correlation coefficients, all *P <* 0.001). The most common enterotypes of the 94 participants were in the following order: B (60%), A (28%), and C (13%). The distribution of enterotypes was similar between the barley groups (B > A > C) and tended to be in the positive direction of PCoA2 in the high barley group, but the values were not significant (correlation coefficient, *P =* 0.08, Fig. S1, see Additional file 1).

### Microbiome

Alpha-diversity did not significantly differ between the barley groups (Table S3, see Additional file 1). The high barley group had a higher abundance of *Bifidobacterium* (*P =* 0.01), *Collinsella* (*P =* 0.03), *Butyricicoccus* (*P =* 0.002), *Dialister* (*P =* 0.04), and *Ruminococcus* 2 (*P =* 0.04) without FDR adjustment (Table 3, Table S4, see Additional file 1). *Subdoligranulum* (*P* = 0.08), *Anaerostipes* (*P* = 0.0502), *Acidaminococcus* (*P* = 0.07), and Ruminococcaceae UCG-013 (*P* = 0.06) tended to be high in the high barley group. Additionally, considering reproducibility in other analyses (Table S5, see Additional file 1), we selected *Bifidobacterium*, *Collinsella*, *Butyricicoccus*, *Dialister*, and *Ruminococcus* 2 as candidate characteristics genera associated with barley consumption.

**Table 3:** Relative abundance (%) of gut bacterial genera in low and high barley groups

	Low barley (*n* = 47)	High barley (*n* = 47)		
	0–3.5 g/1000 kcal ^a^	3.5–28 g/1000 kcal ^a^		
Genus (relative abundance %)	Median (interquartile range)	Median (interquartile range)	*P *^b^ (crude)	*P *^c^ (FDR)
*Bifidobacterium*	2.73 (0.00, 5.10)	5.61 (0.00, 8.12)	0.01	0.37
*Collinsella*	0.92 (0.00, 1.82)	1.95 (0.00, 2.20)	0.03	0.42
*Subdoligranulum*	1.12 (0.00, 1.80)	1.83 (0.00, 2.32)	0.08	0.44
*Anaerostipes*	0.89 (0.02, 1.28)	1.06 (0.07, 1.92)	0.0502	0.42
*Butyricicoccus*	0.43 (0.00, 0.46)	0.65 (0.12, 0.65)	0.002	0.09
Ruminococcaceae UCG-013	0.09 (0.00, 0.27)	0.22 (0.00, 0.34)	0.06	0.44
*Dialister*	0.00 (0.00, 0.31)	0.07 (0.00, 0.43)	0.04	0.42
*Acidaminococcus*	0.00 (0.00, 0.30)	0.03 (0.00, 0.66)	0.07	0.44
*Ruminococcus* 2	0.00 (0.00, 0.37)	0.01 (0.00, 0.86)	0.04	0.42

^a ^Range of barley consumption rate

^b^ Mann-Whitney *U*-test (crude *P*-value)

^c^ Mann-Whitney *U*-test adjusted with the FDR (false discovery rate) method

### ﻿Multiple regression analyses of all participants

We next assessed associations between barley and *Bifidobacterium*, *Butyricicoccus*, *Collinsella*, *Dialister*, and *Ruminococcus* 2 using multiple regression analyses (Table 4, Table S6, see Additional file 1). The results of models 1 and 2 are shown in Table 4, and the results of models 1–3 are shown in Table S6 (see Additional file 1) with detailed data. *Bifidobacterium* had a consistent relationship with barley, and *Butyricicoccus* had a relationship in model 1. Other bacteria had no significant relationship with barley.

**Table 4:** Association between the intestinal bacteria and barley consumption^a^ by multivariate linear regression analyses

	Crude ^b^		Model 1 ^c^		Model 2 ^d^	
Genus	r (SE)	*P*	R (SE)	*P*	R (SE)	*P*
*Bifidobacterium*	2.52 (0.70)	0.012	2.52 (1.00)	0.012	2.61 (1.03)	0.012
*Butyricicoccus*	0.11 (0.05)	0.03	0.11 (0.05)	0.03	0.08 (0.05)	0.102
*Collinsella*	0.23 (0.30)	0.45	0.27 (0.30)	0.38	0.26 (0.31)	0.41
*Dialister*	0.08 (0.09)	0.37	0.10 (0.09)	0.24	0.08 (0.09)	0.41
*Ruminococcus* 2	0.10 (0.15)	0.47	0.10 (0.15)	0.53	0.11 (0.16)	0.48

^a ^The barley consumption group 0: low barley group (0–3.5 g/1000 kcal), 1: high barley group (3.5–28 g/1000 kcal)

^b ^Crude [r (SE)]: Coefficients of a single linear regression model

^c ^Model 1 [R (SE)]: Adjusted with sex, age, risk of diabetes, dyslipidemia, and hypertension

^d ^Model 2 [R (SE)]: In addition to model 1, adjusted with an consumption of cereal, sugar and sweetener, legume, and beverage

### Network analysis of gut bacteria and barley 

Figure S2 (see Additional file 1) shows the results of the network analysis. *Bifidobacterium*, *Butyricicoccus*, *Ruminococcus* 2, Ruminococcaceae UCG-013, *Lachnospira*, and *Tyzzerella* 3 were directly associated with barley consumption, and except for *Ruminococcus* 2, they were classified in the same community group. *Anaerostipes* was not directly associated with barley consumption but was associated via *Lachnospira*, Ruminococcaceae UCG-13, or *Tyzzerella* 3 with barley consumption. In addition, *Anaerostipes* belonged to the same community as barley, *Butyricicoccus*, and *Bifidobacterium*. *Ruminococcus* 2 was directly associated with barley but did not belong to the same community group as barley, *Butyricicoccus*, *Bifidobacterium*, and *Anaerostipes*. Finally, *Dialister* and *Collinsella* seemed to have no relationship with barley because they were positioned far from barley and classified in different groups.

## Discussion

We identified the characteristics of the gut bacteria in a Japanese population that consumes barley. Previous studies reported that the soluble fiber in barley increases the prevalence of intestinal bacteria, such as *Prevotella* 9 and *Blautia*, and alpha-diversity [4, 8]. In our study, *Bifidobacterium* and *Butyricicoccus* abundance tended to increase (Table 4), and we consider these bacteria as specific candidates related to barley consumption in a Japanese population. However, barley consumption might have a limited effect because there were no changes in alpha-diversity.

The consumption of barley tended to be positively related to PCoA2, but this result was not significant (*P =* 0.08, Fig. S1, see Additional file 1). The numbers of each enterotype did not differ between the barley groups. The absolute numbers of enterotype B were 33 (high) and 23 (low). These results suggested that barley consumption slightly shifted the enterotype to B; however, further studies are necessary to confirm this result. Our results of PCoA (Fig. 2b) were similar to those of Arumugam et al. [9], in which the distribution was in a “∧” shape comprising enterotypes A (driven by *Bacteroides*), B (driven by *Blautia*), and C (driven by *Prevotella* 9). However, the influential genus of enterotype B, *Blautia*, was different [9]. Arumugam et al. reported that the driving genera of enterotype A (*Bacteroides*) and C (*Prevotella* 9) were robust between cohorts, but for B it depended on the cohort. Furthermore, *Blautia* was reported as one of the major driving genera [9].

In this study, *Bifidobacterium* and *Butyricicoccus* were identified as characteristic gut bacteria in the high barley group. *Bifidobacterium* was associated with rye-containing beta-glucan consumption in a randomized control trial [17] and was increased with dietary fiber in a systematic review [18]. *Butyricicoccus* was associated with barley in an animal study [19]. Most bacteria relatively close to barley in the network analyses (Fig. S2, see Additional file 1) are known for their characteristics of producing short-chain fatty acids (SCFAs). For example, *Butyricicoccus*, *Subdoligranulum*, *Ruminococcus* 2, and Ruminococcaceae UCG-013 belong to the Ruminococcaceae family, which is well-known for producing SCFAs. *Anaerostipes* belongs to the Lachnospiraceae family, which is also known to produce SCFAs. Many bacteria were more highly correlated with other bacteria than with barley. This suggests that in addition to the effects of barley consumption, there are many bacteria that are indirectly altered by interactions between bacteria. *Bifidobacterium* has been reported to produce SCFAs, such as acetic acid from carbohydrates [20], and are known as characteristic bacteria of Japanese people [1]. *Butyricicoccus* is a prevalent butyric acid producer associated with inflammatory bowel disease prevention [21]. Additionally, SCFAs production from dietary fiber can acidify the gut environment, which alters the microbiome [22]. Therefore, these barley-related bacteria might have a positive influence on the hosts.

Although an association with barley was suggested, the results for *Bifidobacterium* might not be unique to barley, as *Bifidobacterium* abundance has been reported to increase with the consumption of rye [17] and other dietary fiber [18]. *Bifidobacterium* itself is a characteristic bacteria found in abundance in the Japanese population [1]. Therefore, it might increase in abundance with a typical Japanese diet. Most barley consumption is from barley mixed with rice, which is a typical Japanese food pattern, and thus, other dietary factors could be confounding. However, in this study, the association between barley and *Bifidobacterium* was observed even after adjusting for various dietary factors (Table 4, Table S6, see Additional file 1), and therefore, we consider the results robust. Although there are few reports on *Butyricicoccus* in humans, randomized controlled trials have shown that its abundance increases after consuming a Mediterranean diet [23]. Because *Butyricicoccus* is highly capable of fermenting dietary fiber, it is possible that the increase was caused by the whole grains, legumes, and vegetables in the Mediterranean diet, but these details are not known. This is the first study to show an association between barley and *Butyricicoccus* in humans, and it will be interesting to see whether the effect was specific to barley or this cohort. However, because the association between barley and *Butyricicoccus* was found only in model 2, confounding by other dietary factors cannot be ruled out, limiting the interpretation of the present results.

This study has several limitations. First, the participants were employees of a company that manufactures barley products; therefore, the participants would most likely consume more barley than the general population. The possibility of other confounding factors such as dietary habits cannot be completely excluded; thus, the effects of barley could be overestimated. However, the use of this population is appropriate for the following reason. In general, only approximately 15% of the Japanese population consumes barley regularly based on consumer research data, with an average daily consumption of less than 1.0 g (estimate based on government statistics). However, the average consumption of this cohort was as high as 4.6 g, and the range was 0–28 g. Therefore, this is a reasonable population to use for the evaluation of the effects of barley consumption. Second, a detailed barley questionnaire might influence the participants’ dietary and lifestyle habits. Although the participants were instructed not to change their dietary and lifestyle habits before and after the medical check-up, the possibility of bias remaining cannot be denied. Finally, we did not measure SCFAs or microbiome functions, which restricts the usefulness of our results. A follow-up intervention study with another cohort is warranted to overcome these limitations. In conclusion, barley might change the intestinal bacteria of the Japanese population. We selected *Bifidobacterium* and *Butyricicoccus* as candidate characteristic genera indicating barley consumption.

## Supplementary Information

**Additional file 1: Table S1**. Criterion of risk group of diabetes, hypertension, and dislipidemia. **Table S2**. Comparison of diet between low barley group and high barley group on cross-sectional study (*n* 94). **Table S3**. Alpha-diversity, compared low barley with high barley group on cross-sectional study (aged 19–65 years in 2018, Japan). **Table S4**. Relative abundance(%) of the frequent 50 genera, compared low barley with high barley group on cross-sectional study (aged 19–65 years in 2018, Japan). **Table S5**. Results of Multiple regression analysis (dichotomy and continuous) and Kendall rank correlation between barley consumption (g/1000 kcal) and intestinal bacterias on cross-sectional study (aged 19–65 years in 2018, Japan). **Table S6**. Association between the relative abundance of intestinal bacteria and barley intake group (0: low, 1: high) by multivariate linear regression analyses (*N* = 236). **Fig. S1**. PCoA result. Color means high or low consumption of barley (*P* = 0.08). The arrow means high barley group (*n* = 94, aged 19–65 years, Japan, 2018). **Fig. S2**. The result of network analysis. Line means correlation |*r*| > 0.15 (Kendall rank correlation test).

## Data Availability

The datasets supporting the conclusions of this article are included within the article (and its additional file 1). Other data described in the manuscript, code book, and analytic code will be made available from corresponding author upon reasonable request.

## Abbreviations

VIF: Variance inflation factor
PCoA: Principal coordinate analysis
FDA: False discovery rate
SCFA: Short-chain fatty acid
