# Supplementary material for: Relationships between barley consumption and gut microbiome characteristics in a healthy Japanese population: a cross-sectional study
Source: BMC Nutr. 2022 Mar 14;8:23. doi: 10.1186/s40795-022-00500-3 (PMC8919566; doi:10.1186/s40795-022-00500-3)
Supplement: Supplementary file 1 — Additional file 1: Table S1. Criterion of risk group of diabetes, hypertension, and dislipidemia. Table S2. Comparison of diet between low barley group and high barley group on cross-sectional study (n 94). Table S3. Alpha-diversity, compared low barley with high barley group on cross-sectional study (aged 19–65 years in 2018, Japan). Table S4. Relative abundance(%) of the frequent 50 genera, compared low barley with high barley group on cross-sectional study (aged 19–65 years in 2018, Japan). Table S5. Results of Multiple regression analysis (dichotomy and continuous) and Kendall rank correlation between barley consumption (g/1000 kcal) and intestinal bacterias on cross-sectional study (aged 19–65 years in 2018, Japan). Table S6. Association between the relative abundance of intestinal bacteria and barley intake group (0: low, 1: high) by multivariate linear regression analyses (N = 236). Fig. S1. PCoA result. Color means high or low consumption of barley (P = 0.08). The arrow means high barley group (n = 94, aged 19–65 years, Japan, 2018). Fig. S2. The result of network analysis. Line means correlation |r| > 0.15 (Kendall rank correlation test). [file 40795_2022_500_MOESM1_ESM.pdf]

Table S1 Criterion of risk group of diabetes, hypertension, and dislipidemia.

Variable	*N* (Male)	Low barley (*N* = 119)	High barley (*N* = 117)	Criterion
Diabetes risk				
Risk 2	8 (8)	3	5	HbA1c >= 6.5% or Fasting blood glucose >= 126 mg/dL or Under medication
Risk 1	5 (5)	4	1	Who were not Risk 2, and Fasting blood glucose >= 110 mg/dL
Risk 0	223 (161)	110	113	Who were neither Risk 1 nor Risk 2
Hypertension risk				
Risk 2	46 (43)	20	26	SBP >= 140 mmHg or DBP >= 90 mmHg or Under medication
Risk 1	20 (16)	11	9	Who were not Risk 2, and either SBP >= 130 mgHg or DBP >= 85 mgHg
Risk 0	170 (115)	86	84	Who were neither Risk 1 nor Risk 2
Dislipidemia risk				
Risk 2	97 (84)	46	51	TG >= 150 mg/dL or HDL-cholesterol < 40 mg/dL or LDL-cholesterol >= 140 mg/dL or Under medication
Risk 1	31 (25)	17	14	Who were not Risk 2, and LDL-cholesterol >= 120 mg/dL
Risk 0	108 (65)	54	54	Who were neither Risk 1 nor Risk 2

Risk 2 means disease, Risk 1 means border line, and Risk 0 means healthy participants.

Range of barley consumption; Low barley: 0-3.5 g/1000 kcal, High barley: 3.6-28 g/1000 kcal

Table S2 Comparison of diet between low barley group and high barley group on cross-sectional study (*n* 94).

Variable (g/1000 kcal)	Low barley (*n* = 47) 0-3.5 g/1000 kcal[1]	High barley (*n* = 47) 3.5-28 g/1000 kcal[1]	*P* [2]	Model 2[3]	Model 3[3]
Category					
Cereal	213 ± 55	236 ± 58	0.048	O	O
Potato	19 ± 16	21 ± 14	0.40		
Sugar and sweetener	3.9 ± 3.7	2.4 ± 2.4	0.03	O	O
Legume	20 ± 15	27 ± 20	0.056	O	O
Green vegetable	37 ± 24	44 ± 31	0.25		O
Other vegetable [4]	62 ± 29	72 ± 45	0.20		O
Fruit	36 ± 32	35 ± 30	0.87		
Fish	32 ± 15	31 ± 16	0.74		O
Meat	48 ± 26	45 ± 15	0.47		
Egg	18 ± 10	20 ± 13	0.45		
Milk	54 ± 43	48 ± 44	0.49		
Oil	6.9 ± 2.2	7.2 ± 2.2	0.53		
Confectionery	26 ± 20	25 ± 17	0.81		O
Beverage [4]	439 ± 271	335 ± 226	0.047	O	O
Seasoning and spice [4]	128 ± 45	135 ± 70	0.57		
In detail					
Rice	141 ± 54	173 ± 61	0.007		
Natto	3.6 ± 4.2	10.4 ± 12.0	<0.001		
Green leafy vegetables	11 ± 10	17 ± 17	0.04		
Carrot and Pumpkin	6.7 ± 5.9	10.1 ± 8.4	0.03		
Seaweed	3.5 ± 2.8	4.0 ± 3.9	0.51		
Mushroom	4.4 ± 2.5	5.0 ± 4.1	0.37		
Boiled fish	25 ± 28	15 ± 16	0.052		
Japanese confectionery	3.8 ± 3.4	2.8 ± 2.5	0.08		

Ice cream	18 ± 19	12 ± 12	0.08
Coffee	136 ± 113	84 ± 91	0.015
Cola	67 ± 65	37 ± 50	0.013

Data are shown as Means ± SD
1) Range of barley consumption.
2) Student's *t* -test
3) Model variables were used as the independent variables on tha multiple regression analyses.
4) Other vegetable include mushroom and seaweed.
Beverage include fruit or vegetable drink.
Seasoning include liquid seasoning.

Table S3 Alpha-diversity, compared low barley with high barley group on cross-sectional study (aged 19-65 years in 2018, Japan).

	Low barley (*n* = 47) range: 0-3.5 g/1000 kcal	High barley (*n* = 47) range: 3.5-28 g/1000 kcal		
Variable	Median [Interquartile range]	Median [Interquartile range]	P_{crude} [1]	P_{FDR} [2]
Observed	561 [482, 649]	549 [500, 628]	0.86	0.87
Chao1	1146 [919, 1290]	1170 [971, 1419]	0.41	0.68
Shannon	3.77 [3.46, 3.95]	3.77 [3.57, 4.14]	0.38	0.68
Simpson	0.94 [0.90, 0.95]	0.94 [0.92, 0.96]	0.21	0.68
Fisher	128 [106, 156]	125 [111, 149]	0.87	0.87

[1] Compared low and high barley groups using Mann-Whitney *U*-test (crude *P* value)

[2] Compared low and high barley groups using Mann-Whitney *U*-test adjusted with FDR method

Table S4 Relative abundance(%) of the frequent 50 genera, compared low barley with high barley group on cross-sectional study (aged 19-65 years in 2018, Japan).

	Low barley (*n* = 47) range: 0-3.5 g/1000 kcal	High barley (*n* = 47) range: 3.5-28 g/1000 kcal		
Genus	Median [Interquartile range]	Median [Interquartile range]	P_{clude} [1]	P_{FDR} [2]
Bacteroides	31.27 [0.01, 28.98]	29.28 [1.71, 27.52]	0.50	0.79
Blautia	6.15 [0.78, 7.48]	5.81 [1.63, 6.67]	0.64	0.85
Bifidobacterium	2.73 [0.00, 5.10]	5.61 [0.00, 8.12]	0.01	0.37
Faecalibacterium	5.76 [0.00, 6.02]	6.08 [0.00, 6.34]	0.54	0.79
Prevotella 9	0.01 [0.00, 6.96]	0.00 [0.00, 3.90]	0.20	0.59
Eubacterium rectale group	1.10 [0.00, 2.28]	1.56 [0.00, 2.62]	0.73	0.87
Parabacteroides	2.28 [0.00, 2.62]	1.64 [0.00, 1.96]	0.18	0.59
Subdoligranulum	1.12 [0.00, 1.80]	1.83 [0.00, 2.32]	0.08	0.44
Collinsella	0.92 [0.00, 1.82]	1.95 [0.00, 2.20]	0.03	0.42
Sutterella	1.41 [0.00, 1.82]	1.43 [0.00, 2.02]	0.99	0.99
Megamonas	0.00 [0.00, 3.03]	0.00 [0.00, 0.76]	0.20	0.59
Ruminococcus torques group	1.27 [0.03, 1.67]	1.09 [0.01, 1.73]	0.36	0.72
Anaerostipes	0.89 [0.02, 1.28]	1.06 [0.07, 1.92]	0.0502	0.42
Lachnoclostridium	1.22 [0.19, 1.69]	1.23 [0.04, 1.48]	0.53	0.79
Fusicatenibacter	1.41 [0.00, 1.49]	1.00 [0.00, 1.40]	0.83	0.90
Fusobacterium	0.00 [0.00, 1.14]	0.00 [0.00, 1.65]	0.91	0.95
Eubacterium hallii group	0.97 [0.00, 1.28]	0.78 [0.00, 0.97]	0.60	0.83
Alistipes	0.21 [0.00, 0.82]	0.40 [0.00, 1.30]	0.34	0.72
Lachnospira	0.41 [0.00, 0.78]	0.62 [0.00, 1.10]	0.22	0.59
Prevotella 2	0.00 [0.00, 0.34]	0.00 [0.00, 1.46]	0.99	0.99
Megasphaera	0.00 [0.00, 0.65]	0.00 [0.00, 1.15]	0.28	0.66
Roseburia	0.66 [0.00, 1.04]	0.40 [0.00, 0.72]	0.18	0.59

Veillonella	0.03 [0.00, 1.06]	0.03 [0.00, 0.70]	0.47	0.79
Phascolarctobacterium	0.01 [0.00, 0.79]	0.00 [0.00, 0.70]	0.58	0.83
Escherichia Shigella	0.07 [0.00, 0.72]	0.05 [0.00, 0.75]	0.73	0.87
Lachnospiraceae family uncultured	0.49 [0.01, 0.72]	0.59 [0.00, 0.70]	0.91	0.95
Alloprevotella	0.00 [0.00, 0.56]	0.00 [0.00, 0.74]	0.77	0.89
Dorea	0.40 [0.00, 0.81]	0.35 [0.00, 0.43]	0.32	0.72
Ruminococcus 2	0.00 [0.00, 0.37]	0.01 [0.00, 0.86]	0.04	0.42
Butyricicoccus	0.43 [0.00, 0.46]	0.65 [0.12, 0.65]	0.00	0.09
Lachnospiraceae UCG-008	0.52 [0.00, 0.57]	0.32 [0.00, 0.48]	0.81	0.90
Ruminococcus 1	0.01 [0.00, 0.28]	0.01 [0.00, 0.69]	0.34	0.72
Acidaminococcus	0.00 [0.00, 0.30]	0.03 [0.00, 0.66]	0.07	0.44
Ruminococcaceae family uncultured	0.23 [0.00, 0.42]	0.24 [0.00, 0.54]	0.64	0.85
Parasutterella	0.02 [0.00, 0.38]	0.02 [0.00, 0.57]	0.50	0.79
Streptococcus	0.12 [0.00, 0.52]	0.20 [0.00, 0.43]	0.50	0.79
Mitsuokella	0.00 [0.00, 0.59]	0.00 [0.00, 0.35]	0.38	0.73
Prevotellaceae NK3B31 group	0.00 [0.00, 0.48]	0.00 [0.00, 0.28]	0.71	0.87
Dialister	0.00 [0.00, 0.31]	0.07 [0.00, 0.43]	0.04	0.42
Ruminiclostridium 5	0.12 [0.00, 0.36]	0.15 [0.01, 0.32]	0.66	0.85
Ruminococcaceae UCG-013	0.09 [0.00, 0.27]	0.22 [0.00, 0.34]	0.06	0.44
Barnesiella	0.00 [0.00, 0.23]	0.05 [0.00, 0.36]	0.22	0.59
Ruminococcaceae UCG-002	0.00 [0.00, 0.17]	0.01 [0.00, 0.39]	0.19	0.59
Rhodospirillaceae family uncultured	0.00 [0.00, 0.26]	0.00 [0.00, 0.27]	0.43	0.79
Ruminococcus gauvreauii group	0.00 [0.00, 0.23]	0.00 [0.00, 0.25]	0.79	0.90
Paraprevotella	0.00 [0.00, 0.31]	0.00 [0.00, 0.17]	0.20	0.59
Odoribacter	0.04 [0.00, 0.21]	0.10 [0.00, 0.24]	0.48	0.79
Eubacterium coprostanoligenes group	0.00 [0.00, 0.14]	0.01 [0.00, 0.30]	0.21	0.59
Bilophila	0.10 [0.00, 0.18]	0.14 [0.00, 0.25]	0.24	0.60

Tyzzerella 3	0.00 [0.00, 0.10]	0.00 [0.00, 0.29]	0.22	0.59

[1] Compared low and high barley groups using Mann-Whitney *U* -test (crude *P* value)

[2] Compared low and high barley groups using Mann-Whitney *U* -test adjusted with FDR method

Table S5 Results of Multiple regression analysis (dichotomy and continuous) and Kendall rank correlation between barley consumption (g/1000 kcal) and intestinal bacterias on cross-sectional study (aged 19-65 years in 2018, Japan).

	Multiple regression (Barley:dichotomy)[1)]			Multiple regression (Barley:continuous)[2)]			Kendall rank correlation[3)]	
Genus	Estimate	SE	*P* value	Estimate	SE	*P* value	Estimate	*P* value
Bacteroides	-1.51	3.15	0.63	-0.20	0.34	0.57	-0.05	0.48
Blautia	-0.85	0.85	0.32	-0.06	0.09	0.50	-0.05	0.47
Bifidobacterium	2.78	1.63	0.09	0.36	0.18	0.04	0.17	0.01
Faecalibacterium	-0.21	1.09	0.85	-0.14	0.12	0.23	0.07	0.32
Prevotella 9	-2.60	2.91	0.38	-0.25	0.32	0.44	-0.09	0.27
Eubacterium rectale group	0.55	0.58	0.34	0.06	0.06	0.37	0.02	0.80
Parabacteroides	-0.65	0.39	0.10	-0.04	0.04	0.35	-0.11	0.12
Subdoligranulum	0.43	0.46	0.36	-0.03	0.05	0.57	0.06	0.38
Collinsella	0.50	0.52	0.34	0.08	0.06	0.15	0.12	0.10
Sutterella	0.24	0.44	0.59	-0.04	0.05	0.42	-0.03	0.67
Megamonas	-1.87	1.15	0.11	-0.19	0.12	0.13	-0.11	0.19
Ruminococcus torques group	0.16	0.41	0.70	0.00	0.04	0.94	-0.05	0.45
Anaerostipes	0.71	0.36	0.05	0.04	0.04	0.36	0.08	0.25
Lachnoclostridium	-0.17	0.27	0.54	0.00	0.03	0.96	-0.04	0.54
Fusicatenibacter	-0.06	0.27	0.82	-0.02	0.03	0.46	-0.02	0.80
Fusobacterium	0.74	0.85	0.39	0.05	0.09	0.61	-0.03	0.72
Eubacterium hallii group	-0.42	0.24	0.08	-0.02	0.03	0.50	0.00	0.98
Alistipes	0.29	0.37	0.44	0.00	0.04	1.00	0.05	0.44
Lachnospira	0.23	0.25	0.36	0.03	0.03	0.26	0.16	0.03

Prevotella 2	1.41	0.64	0.03	0.03	0.07	0.68	-0.01	0.90
Megasphaera	0.74	0.60	0.22	0.28	0.06	<0.001	0.06	0.43
Roseburia	-0.40	0.22	0.08	-0.03	0.02	0.16	-0.07	0.32
Veillonella	-0.37	0.44	0.40	-0.01	0.05	0.86	0.14	0.07
Phascolarctobacterium	-0.19	0.22	0.39	-0.01	0.02	0.66	0.00	0.96
Escherichia Shigella	0.06	0.47	0.90	0.06	0.05	0.26	0.03	0.69
Lachnospiraceae family uncultured	0.02	0.14	0.90	0.04	0.01	0.01	0.07	0.31
Alloprevotella	0.18	0.56	0.75	-0.04	0.06	0.56	-0.06	0.49
Dorea	-0.32	0.19	0.10	-0.02	0.02	0.39	-0.06	0.36
Ruminococcus 2	0.45	0.23	0.06	0.02	0.03	0.51	0.15	0.049
Butyricicoccus	0.17	0.07	0.02	0.02	0.01	0.052	0.21	0.003
Lachnospiraceae UCG-008	-0.11	0.11	0.32	-0.01	0.01	0.60	0.01	0.93
Ruminococcus 1	0.36	0.21	0.08	0.03	0.02	0.18	0.10	0.20
Acidaminococcus	0.37	0.20	0.07	-0.01	0.02	0.82	0.08	0.29
Ruminococcaceae family uncultured	0.03	0.13	0.81	0.02	0.01	0.15	0.01	0.85
Parasutterella	0.18	0.20	0.38	0.06	0.02	0.01	0.08	0.27
Streptococcus	-0.11	0.17	0.53	0.00	0.02	0.79	0.07	0.32
Mitsuokella	-0.21	0.45	0.65	0.00	0.05	0.93	0.02	0.84
Prevotellaceae NK3B31 group	-0.34	0.41	0.41	-0.03	0.04	0.53	0.00	0.99
Dialister	0.17	0.13	0.19	0.00	0.01	0.92	0.08	0.27
Ruminiclostridium 5	-0.04	0.11	0.72	0.00	0.01	0.95	0.01	0.89
Ruminococcaceae UCG-013	0.06	0.08	0.49	0.01	0.01	0.27	0.17	0.02
Barnesiella	0.09	0.12	0.46	0.01	0.01	0.63	0.08	0.29

Ruminococcaceae UCG-002	0.14	0.13	0.25	0.00	0.01	0.98	0.07	0.38
Rhodospirillaceae family uncultured	0.02	0.21	0.92	0.01	0.02	0.64	-0.09	0.25
Ruminococcus gauvreauii group	0.01	0.11	0.93	-0.01	0.01	0.38	-0.01	0.93
Paraprevotella	-0.15	0.13	0.23	0.00	0.01	0.86	-0.13	0.11
Odoribacter	0.01	0.08	0.87	0.00	0.01	0.88	0.02	0.76
Eubacterium coprostanoligenes group	0.13	0.10	0.21	0.02	0.01	0.03	0.07	0.38
Bilophila	0.05	0.05	0.29	0.00	0.01	0.46	0.06	0.40
Tyzzerella 3	0.19	0.13	0.16	0.06	0.01	0.00	0.16	0.04

[1)] Results of multiple regression analysis. Genus levels of microbiome relative abundance (%) were used as the dependent variable, barley consumption rate (0="Low barley group", 1="High barley group"), age (years), and sex (0="female", 1="male") were used as the independent variable. Estimate: Linear regression coefficient, SE: standard error.

[2)] Results of multiple regression analysis. Genus levels of microbiome relative abundance (%) were used as the dependent variable, barley consumption rate (g/1000 kcal), age (years), and sex (0="female", 1="male") were used as the independent variable. Estimate: Linear regression coefficient, SE: standard error.

[3)] Results of correlation test between barley consumption rate (g/1000 kcal) and genus levels of intestinal bacteria relative abundance (%) using Kendall method. Estimate: Kendall's correlation coefficient.

Table S6 Association between the relative abundance of intestinal bacteria and barley intake group (0: low, 1: high)[1] by multivariate linear regression analyses (*N* = 236).

	Model 1			Model 2			Model 3		
	R	SE	*P* value	R	SE	*P* value	R	SE	*P* value
Bifidobacterium									
Barley (0: low, 1: high)[1]	2.52	1.00	0.012 *	2.61	1.03	0.012 *	2.59	1.03	0.013 *
Sex (0: female, 1: male)	-1.31	1.20	0.28	-0.79	1.24	0.52	-1.22	1.33	0.36
Age (10 years)	0.36	0.49	0.46	0.25	0.51	0.63	0.35	0.51	0.49
Risk of diabetes (0–2)	0.45	1.32	0.73	0.79	1.34	0.55	0.96	1.34	0.48
Risk of dyslipidemia (0–2)	-0.17	0.60	0.77	-0.14	0.60	0.81	-0.09	0.60	0.87
Risk of hypertension (0–2)	-1.31	0.71	0.07 #	-1.03	0.73	0.16	-0.98	0.73	0.18
Cereal (g/1000 kcal)				-0.01	0.01	0.44	-0.02	0.01	0.101
Sugar and sweetner (g/1000 kcal)				0.26	0.13	0.04 *	0.27	0.13	0.03 *
Legume (g/1000 kcal)				0.00	0.03	0.95	0.01	0.03	0.62
Beverage (g/1000 kcal)				0.00	0.00	0.06 #	-0.01	0.00	0.009 **
Green vegetable (g/1000 kcal)							-0.03	0.02	0.23
Other vegetable (g/1000 kcal)							-0.01	0.02	0.68
Fish (g/1000 kcal)							-0.06	0.03	0.04 *
Confectionery (g/1000 kcal)							-0.02	0.04	0.60
Butyricicoccus									
Barley (0: low, 1: high)[1]	0.11	0.05	0.03 *	0.08	0.05	0.102	0.08	0.05	0.13
Sex (0: female, 1: male)	0.02	0.06	0.73	0.03	0.06	0.64	0.07	0.07	0.32
Age (10 years)	0.01	0.02	0.75	0.01	0.03	0.60	0.01	0.03	0.73
Risk of diabetes (0–2)	0.02	0.07	0.70	0.03	0.07	0.64	0.04	0.07	0.54
Risk of dyslipidemia (0–2)	-0.02	0.03	0.44	-0.02	0.03	0.41	-0.03	0.03	0.39
Risk of hypertension (0–2)	-0.01	0.04	0.81	-0.01	0.04	0.72	0.00	0.04	0.95
Cereal (g/1000 kcal)				0.00	0.00	0.43	0.00	0.00	0.11
Sugar and sweetner (g/1000 kcal)				0.00	0.01	0.78	0.00	0.01	0.76

Legume (g/1000 kcal)				0.00	0.00	0.16	0.00	0.00	0.17
Beverage (g/1000 kcal)				0.00	0.00	0.34	0.00	0.00	0.92
Green vegetable (g/1000 kcal)							0.00	0.00	0.22
Other vegetable (g/1000 kcal)							0.00	0.00	0.44
Fish (g/1000 kcal)							0.00	0.00	0.99
Confectionery (g/1000 kcal)							0.00	0.00	0.15
Collinsella									
Barley (0: low, 1: high)[1]	0.27	0.30	0.37	0.26	0.31	0.41	0.15	0.31	0.63
Sex (0: female, 1: male)	0.44	0.36	0.23	0.49	0.38	0.20	0.41	0.41	0.31
Age (10 years)	-0.09	0.15	0.52	-0.11	0.15	0.49	-0.09	0.16	0.56
Risk of diabetes (0–2)	-0.26	0.40	0.51	-0.24	0.41	0.56	-0.17	0.41	0.68
Risk of dyslipidemia (0–2)	0.03	0.18	0.86	0.04	0.18	0.84	0.04	0.18	0.83
Risk of hypertension (0–2)	-0.06	0.21	0.77	-0.05	0.22	0.81	0.04	0.22	0.87
Cereal (g/1000 kcal)				0.00	0.00	0.70	0.00	0.00	0.75
Sugar and sweetner (g/1000 kcal)				0.00	0.04	0.93	0.00	0.04	0.97
Legume (g/1000 kcal)				0.00	0.01	0.86	0.00	0.01	0.79
Beverage (g/1000 kcal)				0.00	0.00	0.69	0.00	0.00	0.61
Green vegetable (g/1000 kcal)							0.00	0.01	0.82
Other vegetable (g/1000 kcal)							0.01	0.01	0.08 #
Fish (g/1000 kcal)							-0.02	0.01	0.02 *
Confectionery (g/1000 kcal)							0.00	0.01	0.85
Ruminococcus 2									
Barley (0: low, 1: high)[1]	0.10	0.15	0.53	0.11	0.16	0.48	0.09	0.16	0.58
Sex (0: female, 1: male)	-0.26	0.19	0.16	-0.18	0.19	0.35	-0.12	0.21	0.58
Age (10 years)	0.09	0.07	0.26	0.08	0.08	0.29	0.07	0.08	0.35
Risk of diabetes (0–2)	0.11	0.20	0.60	0.09	0.21	0.65	0.14	0.21	0.50
Risk of dyslipidemia (0–2)	0.00	0.09	1.00	0.02	0.09	0.81	0.03	0.09	0.73
Risk of hypertension (0–2)	-0.11	0.11	0.34	-0.11	0.11	0.32	-0.09	0.11	0.45

Cereal (g/1000 kcal)				0.00	0.00	0.29	0.00	0.00	0.42
Sugar and sweetner (g/1000 kcal)				-0.01	0.02	0.55	-0.01	0.02	0.53
Legume (g/1000 kcal)				-0.01	0.00	0.20	0.00	0.00	0.41
Beverage (g/1000 kcal)				0.00	0.00	0.20	0.00	0.00	0.23
Green vegetable (g/1000 kcal)							0.00	0.00	0.34
Other vegetable (g/1000 kcal)							0.00	0.00	0.37
Fish (g/1000 kcal)							0.00	0.00	0.34
Confectionery (g/1000 kcal)							0.01	0.01	0.33
Dialister									
Barley (0: low, 1: high)[1]	0.10	0.09	0.24	0.08	0.09	0.41	0.06	0.09	0.50
Sex (0: female, 1: male)	0.20	0.11	0.06 #	0.14	0.11	0.19	0.20	0.12	0.098 #
Age (10 years)	-0.03	0.04	0.42	-0.02	0.04	0.64	-0.03	0.05	0.53
Risk of diabetes (0–2)	-0.08	0.12	0.48	-0.10	0.12	0.41	-0.07	0.12	0.54
Risk of dyslipidemia (0–2)	-0.06	0.05	0.27	-0.07	0.05	0.19	-0.07	0.05	0.22
Risk of hypertension (0–2)	-0.04	0.06	0.54	-0.06	0.06	0.37	-0.04	0.07	0.53
Cereal (g/1000 kcal)				0.00	0.00	0.12	0.00	0.00	0.06 #
Sugar and sweetner (g/1000 kcal)				-0.01	0.01	0.25	-0.01	0.01	0.23
Legume (g/1000 kcal)				0.00	0.00	0.29	0.00	0.00	0.20
Beverage (g/1000 kcal)				0.00	0.00	0.09 #	0.00	0.00	0.055 #
Green vegetable (g/1000 kcal)							0.00	0.00	0.85
Other vegetable (g/1000 kcal)							0.00	0.00	0.35
Fish (g/1000 kcal)							0.00	0.00	0.70
Confectionery (g/1000 kcal)							0.00	0.00	0.19

[1]The range of barley intake is 0-3.5 g/1000 kcal in low, 3.5-28 g/1000 kcal in high.
Character means ** : P < 0.01, *: P < 0.05, #: P < 0.1
Model 1: Adjusted with sex, age, risk of diabetes, dyslipidemia, and hypertension.
Model 2: In addition to model 1, adjusted with an intake of cereal, sugar and sweetener, legume, and beverage.
Model 3: In addition to model 2, adjusted with green vegetable, other vegetable and confectionery

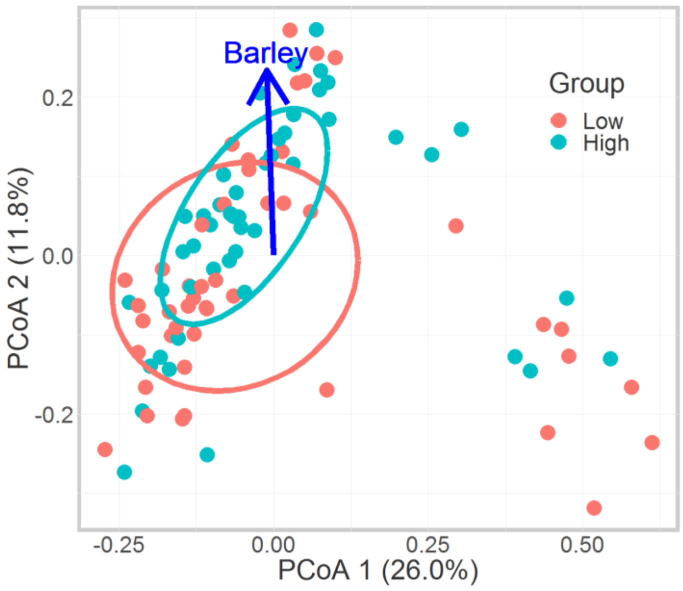

Figure S1 PCoA result. Color means high or low consumption of barley (P = 0.08). The arrow means high barley group (n = 94, aged 19-65 years, Japan, 2018).

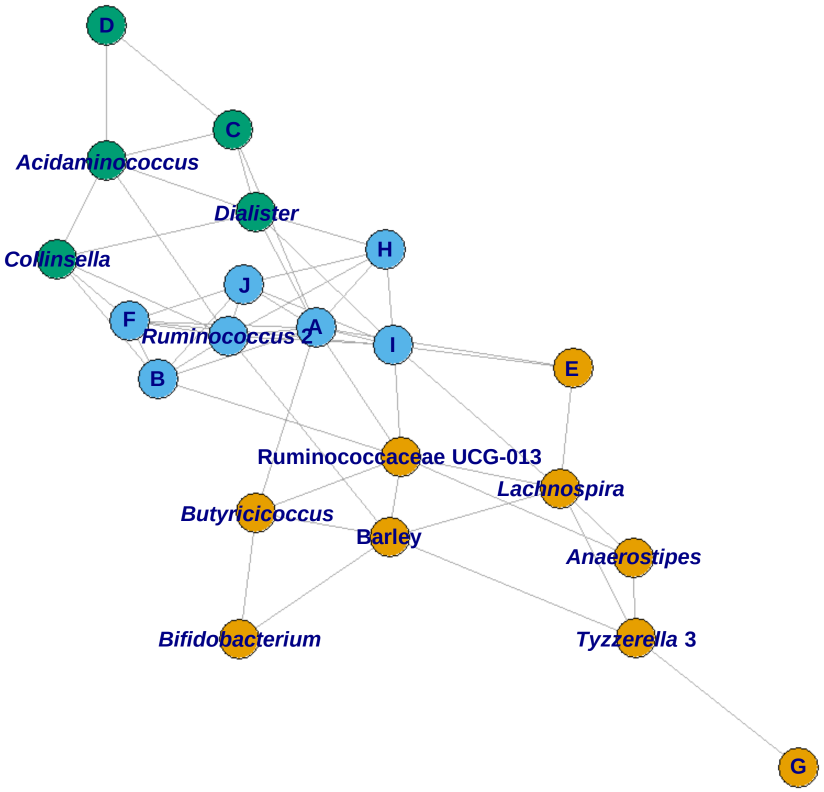

Figure S2 The result of network analysis. Line means correlation |*r*| > 0.15 (Kendall rank correlation test). Colors mean community groups. Described names are those in which the difference between low barley and high barley is *P* < 0.1 (Mann-Whitney *U*-test) (*n* = 94, aged 19-65 years, Japan, 2018).

The bacteria described as characters are as follow; A: *Subdoligranulum*, B: *Eubacterium* hallii group, C: *Prevotella* 2, D: *Megasphaera*, E: *Roseburia*, F: *Veillonella*, G: Lachnospiraceae family uncultured, H: *Dorea*, I: *Ruminococcus* 1, J: *Eubacterium* coprostanoligenes group.
